# Out-of-hours workload among Norwegian general practitioners – an observational study

**DOI:** 10.1186/s12913-020-05773-7

**Published:** 2020-10-14

**Authors:** Ingrid Keilegavlen Rebnord, Tone Morken, Kjell Maartmann-Moe, Steinar Hunskaar

**Affiliations:** 1 National Centre for Emergency Primary Health Care, NORCE Norwegian Research Centre, Aarstadveien 17, NO-5009 Bergen, Norway; 2 The Norwegian Directorate of Health, Oslo, Norway; 3 Department of Global Public Health and Primary Care, University of Bergen, Bergen, Norway

**Keywords:** General practice, Family medicine, Out-of-hours medical care, Primary care, Workload, Survey, Observational study

## Abstract

**Background:**

Repeated studies of working hours among Norwegian regular general practitioners (RGPs) have shown that the average total number of weekly working hours has remained unchanged since 1994 and up until 2014. For both male and female RGPs, the mean total weekly working hours amounted to almost 50 h in 2014.

In recent years, Norwegian RGPs have become increasingly dissatisfied. They experience significantly increased workload without compensation in the form of more doctors or better payment. A study from the Norwegian Directorate of Health in 2018 (the RGP study) showed that Norwegian RGPs worked 55.6 h weekly (median 52.5). 25% of the respondents worked more than 62.2 h weekly.

Based on data from the RGP study we investigated Norwegian RGP’s out-of-hours (OOH) work, how the working time was distributed, and to what extent the OOH work affected the regular working hours.

**Methods:**

In early 2018, an electronic survey was sent to all 4640 RGPs in Norway. Each RGP reported how many minutes that were spent that particular day on various tasks during seven consecutive days. Working time also included additional tasks in the municipality, other professional medical work and OOH primary health care. Differences were analysed by independent t-tests, and regression analyses.

**Results:**

One thousand eighty hundred seventy-six RGPs (40.4%) responded, 640 (34.1%) had registered OOH work. Male RGPs worked on average 1.5 h more doing regular work than did females (*p* = 0.001) and on average 2.3 h more OOH work than females (*p* = 0.079). RGPs with no OOH work registered a mean of 1.0 h more clinical work than RGPs working OOH (*p* = 0.043). There was a large variation in OOH working hours. A linear regression analysis showed that male RGPs and RGPs in rural areas had the heaviest OOH workload.

**Conclusions:**

One in three Norwegian RGPs undertook OOH work during the registration week in the RGP study. OOH work was done in addition to a sizeable regular workload as an RGP. We found small gender differences. OOH work was not compensated with reduced regular RGP work.

## Background

In 2001, Norway introduced a list-based medical primary care service for its inhabitants. All residents have an offer to be on the list of a regular general practitioner (RGP). This ensures continuity in the doctor-patient relationship and more equal healthcare services for all inhabitants. The agreement between the RGPs and the municipality involves the responsibility for the patients on the list but also entails an obligation to participate in the municipal emergency out-of-hours (OOH) services [1]. The way this emergency care is organized varies in the different municipalities due to differences in population density and geography. Large variations in the participation of RGPs in OOH services have been found in previous studies [2–5].

In 2017, approximately 60% of the RGPs participated in OOH work. Many of them worked only part of their full obligations [3, 6]. Older doctors and female doctors in central municipalities participated least. Few doctors did OOH work after the age of 55. These trends have been relatively stable over recent years.

A systematic review in 2006 found that factors related to the profession such as task variation, contact and relationships with colleagues and teaching students often increased job satisfaction On the other hand, employment conditions like low income, too many working hours, administrative burden, and lack of time and recognition were associated with lower job satisfaction [7]. A Norwegian study in 2010 found that the job satisfaction among Norwegian RGPs was high and was rising between 2000 and 2006 [8]. In recent years, increasing dissatisfaction with the workload has been reported among RGPs [9, 10]. The Care Coordination Reform has gradually been implemented since 2012 [11]. This reform aims to improve the collaboration and coordination between primary and secondary health care. The municipalities were supposed to take the responsibility for more patients, avoid referrals to hospital and receive patients from the hospitals at an earlier stage. Each RGP got increased responsibility for the management of each patient but was supposed to be responsible for fewer patients. However, the increase in the number of RGPs has been much lower than anticipated, and fewer additional resources have been added to the scheme [12, 13].

Repeated studies on Norwegian doctors’ working hours showed that, for most doctors, total weekly working hours remained unchanged from 1994 until 2014 [14]. In 2014, the mean number of total working hours was 49.2 among male RGPs and 48.1 among females.

In recent years, there has been an outcry of dissatisfaction among Norwegian RGPs. They experience significantly increased workload without compensation in the form of more doctors or better payment. A study from the Norwegian Directorate of Health in 2018 (the RGP study) showed that Norwegian RGPs on average worked 55.6 h weekly (median 52.5), and 25% worked more than 62.2 h weekly [15, 16].

Based on data from the RGP-study [15] we further investigated the characteristics of RGPs’ working OOH, the distribution of OOH work, how OOH work affects the regular working hours, and several characteristics of RGPs a heavy OOH workload.

## Method

In January 2018, an electronic survey was sent by email to all available RGPs (*n* = 4640) in Norway. The aim was to monitor working hours of RGPs as precisely as possible for one week. The mailing list was based on addresses from Norwegian Healthnet (NHN) and The Norwegian Health Economics Administration (HELFO). Non-responders got reminder emails one and two weeks after the first email was dispatched. In addition to the invitation email, The Norwegian Directorate of Health sent information about the study to all municipalities, and The Norwegian Medical Association sent information to all their RGP members to encourage participation in the study. The study protocol was submitted to and approved by the Ombudsman for Research, Norwegian Centre for Research Data (NSD).

### Survey instrument

The authors designed the questionnaire in Qualtrics software (version 2018 of Qualtrics, copyright© 2018, Provo, UT), and pilot tested it on 30 RGPs. The questionnaire included the following items: Gender, age, whether the participant was an approved specialist in general practice or not, number of days per week in clinical daytime practice, number of years working as a RGP, employment position, number of inhabitants on their RGP list, number of inhabitants in the municipality, and travel distance to the nearest hospital. For each of seven consecutive days, the RGP was to register how many minutes per day they spent on various tasks in the RGP practice and the time spent for additional positions in the municipality or other positions. The doctors were also to register how many hours they spent on duty at the OOH services each day during that week.

We asked about three different types of OOH-work: (1) Working at an OOH clinic, which means that the doctor is present at the clinic throughout the working hours. This kind of duty is common in cities and inter-municipal cooperatives (2); Duty from home parts of the day while responding with turnouts immediately upon urgent inquiries and doing home visits or office consultations when appropriate. This is common in more rural areas with only one GP on duty (3); Being the second doctor on call as support for less experienced doctors or locums. Most inquiries are solved by telephone. This kind of duty is considered less demanding than the two others. Hence, we excluded hours from the third category in some analyses.

“Clinical work” is daytime clinical practice work related to the patient list measured in hours per week. “Regular working hours” is defined as all clinical work, additional work for the municipality or other positions, administration of practice and teaching. “Total working hours” is defined as OOH work in addition to regular working hours.

### Statistical analyses

Descriptive statistics were used, given as mean, median and proportions. To identify differences between groups, independent t-tests and multiple regression analyses were used. *Cohen’s d* (standardized mean difference) was used to measure the effect size between the means. In general: *Cohen’s d* -value of 0.8 is defined as large effect, 0.5 is medium effect and 0.2 is small effect. *Cohen’s d* of 1 indicates that the two groups differ by 1 standard deviation; a *d* of 0.5 indicates they differ by 0.5 standard deviations, and so on. A multiple linear regression analysis was performed to identify factors associated with heavy OOH workload. Different models for selection of variables were tested but we found no differences in R-square between the models, and therefore a stepwise selection was chosen. The level of statistical significance was set at *p* = 0.05. The statistical analyses were performed in IBM SPSS Statistics, version 25.

## Results

The response rate was 40.4% (1876 RGPs). A total of 640 RGPs (34.1%) had also registered work OOH during their registration week.

### Characteristics of all RGPs

48.5% of all respondents were females, compared to 42.0% in the total national RGP population. Mean total working time per week amounted to 55.6 h. Male RGPs worked 57.2 h while females worked 53.9 h (*p* < 0.001) and *Cohen’s d* was 0.16. The difference between male and female RGPs regular work at daytime was 1.5 h per week (p < 0.001) and *Cohen’s d* was 0.12.

### Distribution of the OOH working hours

Table 1 shows the distribution of the different types of OOH work. The main proportion of the RGPs (79.7%) worked at an OOH-clinic, 24.1% worked from home and 24.4% were also on call as a second doctor. Only 8.8% (56) of the RGPs had duties as a second doctor on call only. When estimating the total number of OOH working hours, we excluded the hours worked on call as a second doctor. The minimum value of OOH work was 0.5 h and the maximum value was 168.0 h (all hours in the week). The median value for total OOH working hours was near 10 h.

**Table 1:** Distribution of different types of OOH work

		Work at the OOH-clinic (***n*** = 510) Hours	OOH work on duty from home (***n*** = 154) Hours	OOH work as second doctor on call (***n*** = 156) Hours	Total OOH work, hours as second doctor excluded (***n*** = 584) Hours
**All (mean)**		11.4	19.7	23.1	15.2
**Male**		12.0	22.0	23.1	16.2
**Female**		10.7	16.5	22.5	13.8
**Percentiles**	**10**	6.0	2.0	5.0	6.0
	**25**	7.0	6.5	8.0	7.3
	**50**	8.8	12.5	15.0	9.8
	**75**	15.0	23.0	15.0	16.8
	**90**	20.2	48.0	20.2	28.1

### Characteristics of RGPs working OOH

Among RGPs working OOH, the proportion of males was higher than females (37% vs. 31%). Male RGPs worked on mean average 2.3 h more per week than female RGPs with OOH work but this difference was not significant (*p* = 0.079) and the effect size *Cohen’s d* was 0.15.

In bivariate analysis, the proportion of specialists, older and more experienced RGPs was lower among RGPs working OOH (Table 2). There was also a larger proportion of RGPs working OOH in small municipalities with longer distances to the nearest hospital. Larger list size also seemed to negatively affect participation; the same was found for the participants who were self-employed RGPs.

**Table 2:** Distribution of characteristics of RGPs working OOH and not OOH. *N* = 1876

Variables	Number	RGPs not working OOH (***n*** = 1236) %	RGPs working OOH (***n*** = 640) %	***P*** value
**Gender**				0.005
Female	910	51.0	44.2	
Male	959	49.0	55.8	
**Specialist in general practice**				< 0.001
Yes	1267	74.6	55.3	
No	596	25.4	44.7	
**Experience (number of years as RGP)**				< 0.001
0–2	225	9.2	18.1	
3–5	273	10.2	23.8	
6–10	354	17.4	22.7	
11–15	233	12.0	13.8	
16–25	330	20.2	13.3	
> 25	430	31.0	8.3	
**Age**				< 0.001
< 35	263	10.6	23.0	
35–44	690	30.4	50.0	
45–54	390	21.4	20.0	
> 54	518	37.6	9.0	
**Number of patients on their list**				< 0.001
< 600	72	3.5	4.6	
601–900	316	14.3	22.3	
901–1200	721	35.8	44.5	
1201–1500	567	33.9	24.0	
1501–1800	139	9.6	3.5	
> 1800	43	2.9	1.1	
**Number of inhabitants in the municipality**				< 0.001
< 3000	50	1.6	4.7	
3001–5000	70	2.0	7.1	
5001–10,000	198	8.3	15.3	
10,001–25,000	407	17.9	29.7	
25,001–50,000	376	21.8	17.4	
50,001–100,000	281	16.7	12.2	
> 100,000	471	31.6	13.4	
**Driving distance to nearest hospital**				< 0.001
< 30 min	1372	81.5	58.3	
30 min – 1 h	338	14.5	25.1	
1–2 h	115	3.1	12.2	
> 2 h	39	0.9	4.4	
**Employment position**				< 0.001
Self-employed	1584	90.6	76.1	
Self-employed with bonus agreement	192	7.1	16.6	
Salaried	45	1.6	4.0	
Salaried with bonus agreement	28	0.6	3.3	

A multiple logistic regression analysis showed which RGP characteristics were associated with participation in OOH work. Being a male RGP, long driving distance to the nearest hospital and salaried employment with a bonus agreement were all significantly associated with more OOH work (Table 3). Age more than 54 years and working in cities with more than 100,000 inhabitants were associated with less participation in OOH work.

**Table 3:** Factors associated with working out-of-hours compared to not. Multiple regression analysis. *N* = 1774

Variables	OR	95% CI		***P***-value
**Gender**				
Male (reference)	1.00			
Female	0.53	0.423	0.674	**< 0.001**
**Age**				
< 35 (reference)	1.00			
35–44	1.21	0.839	1.747	0.307
45–54	0.80	0.477	1.357	0.414
> 54	0.18	0.096	0.340	**< 0.001**
**Specialist in general practice**				
Yes (reference)	1.00			
No	1.07	0.777	1.484	0.665
**Experience, number of years as RGP**^**a**^	0.97	0.931	1.001	0.057
**Number of days in clinical practice per week**^**b**^	1.00	0.919	1.095	0.945
**Number of patients at their list**				
< 600 (reference)	1.00			
601–900	1.60	0.850	3.011	0.145
901–1200	1.68	0.897	3.156	0.105
1201–1500	1.54	0.794	3.004	0.200
1501–1800	0.99	0.438	2.258	0.989
> 1800	1.65	0.526	5.078	0.395
**Number of inhabitants in the municipality**				
< 3000 (reference)	1.00			
3001–5000	1.46	0.609	3.502	0.396
5001–10,000	0.97	0.449	2.079	0.930
10,001–25,000	1.25	0.575	2.696	0.578
25,001–50,000	0.69	0.308	1.550	0.370
50,001–100,000	0.72	0.312	1.643	0.431
> 100,000	0.41	0.177	0.932	**0.033**
**Employment position**				
Self-employed (reference)	1.00			
Self-employed with bonus agreement	1.25	0.818	1.898	0.305
Salaried	1.57	0.735	3.340	0.245
Salaried and bonus agreement	4.06	1.320	12.461	**0.014**
**Driving distance to nearest hospital**				
Less than 30 min (reference)	1.00			
30 min – 1 h	1.58	1.161	2.151	**0.004**
1–2 h	5.01	2.881	8.714	**< 0.001**
More than 2 h	3.09	1.317	7.237	**0.010**

^**a**^Continuous variable per year. ^**b**^Continuous variable per half day, minimum 0.5 and maximum 10

**Fig. 1:**
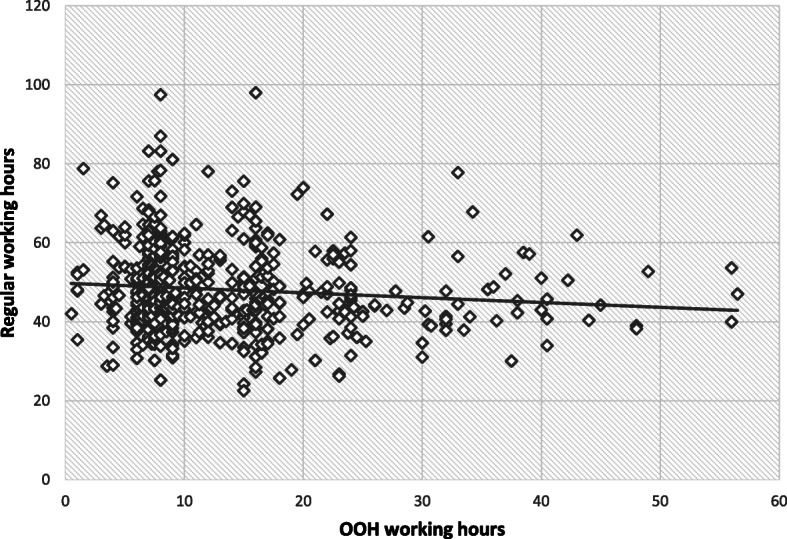
Comparison of regular working time. Scatterplot of paired regular work and OOH work in hours for RGPs working OOH. *N* = 567

There was no significant difference between the regular daytime work among RGPs working or not working OOH. RGPs working OOH had 0.7 h more of regular work during daytime than RGPs not working OOH (*p* = 0.471). Figure 1 shows a scatterplot of paired values of OOH working hours by regular working hours.

The difference in hours of clinical work between the RGPs working OOH and RGPs not working OOH was small (1.0 h per week) but statistically significant (*p* = 0.043). Cohen’s *d* was only 0.10. The difference was primarily explained by the amount of face-to-face contacts (0.7 h more for RGPs with no OOH work, *p* = 0.030, Cohen’s *d* 0.11) and paper work (0.7 h more for RGPs with no OOH work, *p* = 0.002, Cohen’s *d 0.15*). Doctors not working OOH had 0.1 h less home visits per week (*p* = 0.038, Cohen’s *d* 0.10) and 0.2 h less meetings per week (*p* = 0.003, Cohen’s *d* 0.14).

**Fig. 2:**
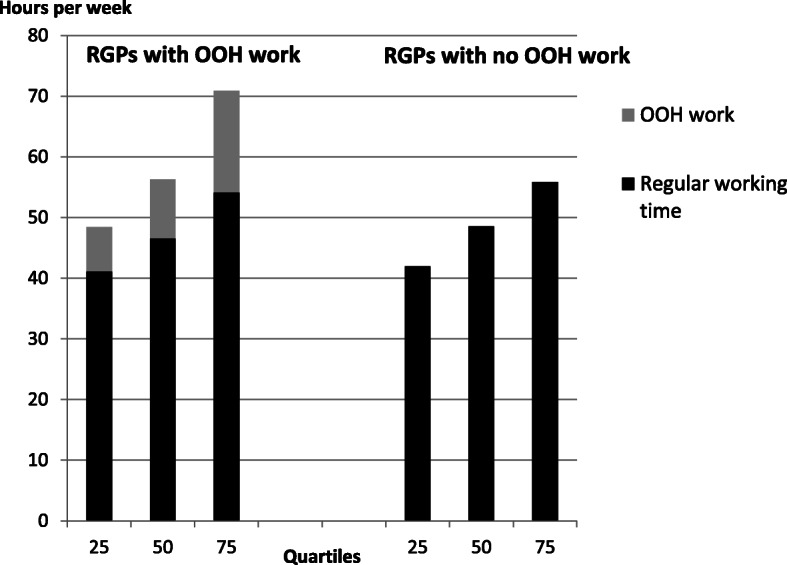
Comparison of total working time. Comparison of total working time per week between RGPs working or not working OOH in quartiles of total working time. *N* = 1876

Figure 2 illustrates the distribution of total working time in quartiles, with OOH work separated from regular working time. We found that in all quartiles, the differences in regular work between the groups were small; it was the OOH work that mainly made the difference in the total working hours, both for those with fewer regular working hours as well as for those with a high number of regular working hours.

### Factors associated with heavy OOH workload

There was a large variation in OOH working hours especially among those working most. To ascertain variables associated with a high number of working hours OOH, a linear regression analysis was performed with possible explanation variables included (Table 4). A stepwise selection method showed that being a male RGP, working in small municipalities and having a long travelling distance to nearest hospital were factors associated with more OOH work per week. Moreover, the RGPs with many hours of OOH work had shorter patient lists.

**Table 4:** Factors associated with heavy workload out-of-hours. *N* = 573

Variables	Standardized Beta (β)	***P***-value
**Gender** (Male = 0 Female = 1)	−0.137	0.001
**Number of inhabitants in the municipality** (7 categories, ref. Table 1)	−0.114	0.025
**Driving distance to nearest hospital** (per 30 min, 4 categories ref. Table 1)	0.211	< 0.001
**Number of patients at their list** (per 300 patient, 6 categories, ref. Table 1)	−0.113	0.010

Linear regression analysis of total working hours OOH exclusive work as second doctor. R square 0.125

## Discussion

During one week in January 2018, every third RGP in Norway was working OOH. OOH work was done in addition to an already heavy workload as an RGP. RGPs working OOH has slightly less clinical daytime practice than those not working OOH. Male RGPs and RGPs from rural areas have the heaviest OOH workload.

### Strengths and limitations

We used an electronic survey because of its clear advantages, e.g. timesaving, cost-effective, no need of data entry. As far as we know, among the Norwegian GPs, all age groups are very well-acquainted with web-based surveys. The response rate of 40.4% is rather low, with a risk of nonresponse bias. However, compared to other studies among physicians, our response rate on an electronic survey is quite good [17]. Generally, physicians have lower response rates than the general public, and different factors that may increase the rate are found in some reviews and trials [17, 18].

The survey was relatively time-consuming to answer since all work-related activity had to be registered continuously for seven consecutive days. The response rate was slightly lower in the oldest age groups. This explains that the female proportion was slightly higher than the national average among RGPs [15]. Despite this small difference, we found our study sample representative with respect to age, gender, list size and proportion of participants who were approved specialists in general practice.

Self-reported working time has some disadvantages. Previous research on RGPs’ workload in Norway was also by self-registration but on smaller numbers of RGPs (*n* = 203) and retrospective [14]. The strength of our study is that all RGPs in Norway got an invitation. The potential degree of over- or under-estimation of working hours in this study is not known. There is a possibility that more hard-working RGPs respond compared to those working less, as the latter may feel they should not ‘spoil’ a desired outcome of heavy workload. On the other hand, experienced RGPs who are used to a heavy workload and those who are comfortable with it may not respond either. OOH work is easier to register than other work tasks that flow more into each other, as the duties are set up with certain hours and paid per hour. Therefore, we assume that the number of hours OOH is correct with a relatively high degree of accuracy.

### Discussion of results

In this study, one of three RGPs worked OOH during one week in January 2018. From other Norwegian studies, we know that more than one third of all RGPs participate in OOH work. In 2017 around 60% of RPGs participated but they had fewer contacts than other doctors at OOH-services and therefore probably take fewer duties [3, 6]. Since our registration was only for one week, there are probably more doctors working OOH less frequently. These RGPs are probably working in larger an OOH-district where many doctors participate. That is why we reckon that the portion of RGPs working OOH is lower in our study sample than in the general RGP population. Our study cannot state the total workload of OOH work for all RGPs but show a mean average for the situation during one normal January week.

Total workload for RGPs is a sum of different tasks. Clinical work associated directly with the patient list is the main task, but OOH work and additional work for the municipality are also compulsory in Norway. Countries that have the same organization as Norway report significant out-of-hours demand and heavy workload in rural areas [19, 20]. The mean total number of working hours for RGPs was 7 h more than found in 2014 [14]. Because of different methods and numbers in the study population, it cannot be concluded that there was an exact increase of 7 h from 2014 until 2018, but our study shows that an increase in the workload is highly likely. Both studies have OOH work included in the mean, and in our study, the mean total number of working hours is higher for both RGPs groups, including those not working OOH. The total workload for the average RGP is some 20 h a week above the recommended working hours in Norway, both for regular work and total work [21].

Mean regular working hours are similar for RGPs both with and without OOH work. It can be compared with GPs in other countries, for example British GPs, i.e. approximately 49 h per week but with large variations. Our study showed that for all RGPs the OOH work is in addition to already more than full-time RGP work at daytime. There was no association between number of regular working hours and OOH working hours, and only minimal reduction in mean regular work at daytime for RGPs with OOH work. There were also minimal differences in time spent on other tasks. This can be explained by the fact that most RGPs have their own personal list and the same duty to work OOH with minor opportunities for flexibility in taking over the work of colleagues.

The small difference in working time between genders in Norway is remarkable. In the United Kingdom, the difference between male and female GPs is estimated to be 6 h for regular work [22] and in Netherland to be 8 h on average [23]. For OOH work we have not found any comparable literature. Our results show that female RGPs have an almost similar workload as males in daytime practice, the gender difference is somewhat greater for OOH (2.3 h compared to 1.6 h). Significantly fewer females participate in OOH work. Especially when the duties are taken from home (rural areas), female RGPs participate less than male RGPs. This is in line with what is known about gender differences generally in working life in Norway. A larger proportion of women work part-time, and this includes highly educated women [24].

Age over 55 years exempts RGPs from OOH work according to the negotiated collective agreement. This fits well with what we found; RGPs above the age of 54 seldom worked OOH. There was a clear tendency that a higher proportion of RGPs in the younger age group participated in OOH work. This may be desired, or may fulfil a requirement to work OOH as part of the specialization course for general practitioners, along with a desire for higher income at the start of the career [25].

The employment position was also associated with participation in OOH work. A higher proportion of salaried GPs or GPs with a bonus agreement participated in OOH work. For small and/or rural municipalities with heavy OOH workloads, different bonus agreement may be offered as a recruitment effort and explains why a salaried position with a bonus agreement was significantly associated with having OOH work.

Different variables were associated with heavy OOH workload and most of them express different conditions in rural areas. Long travelling time to the nearest hospital and small municipalities are two isolated factors associated with heavy OOH workload. We also found that the more hours a RGP worked OOH, the greater the portion of home visit duties. Rurality is associated with OOH home visit duties and shorter patient lists; this is very likely an explanatory factor. We had no information about the centrality of the workplaces. Hence it was not possible to correct for this relationship in the analyses.

To organize emergency primary health care in a rural country such as Norway is challenging, but it is at the same time necessary to provide all citizens with equal health care. Our study has shown that with an increasing workload of regular working hours, there is a risk that fewer RGPs will participate in OOH work. In districts with optional participation, there will be a risk that the RGP’s competence will be lost to the OOH services. In other districts with mandatory participation in OOH work, there will be a risk of recruitment problems if workloads become excessive and the proportion of women among younger doctors increases. For responsible authorities who plan to organize the OOH services, it is important to look at the total workload, so that RGPs are ensured an acceptable total workload in line with other employees who participate in shift work.

### Implications for future research

In this study we investigated self-registered working time during one week. Factors that can provide stability and continuity in primary health care were not mapped. Heavy workload indicates a high risk of unstable physician staffing. With increasing numbers of female medical students, it is necessary to examine what conditions are fundamental to enable newly educated female doctors to thrive and stay in the field of RGPs, especially in rural areas where the OOH workload is heavy.

## Conclusions

During one week, every third RGP in Norway works OOH in addition to their work as RGP. RGPs working OOH do not work less during daytime than RGPs not working OOH. Male RGPs and RGPs from rural areas have the heaviest workload in terms of OOH work. Gender differences are minimal during daytime but more prevalent during OOH work.

## Supplementary information

**Additional file 1.** Qualtrics Survey

## Data Availability

The datasets used during the current study are available from the corresponding author on reasonable request.

## Abbreviations

RGP: Regular general practitioner
OOH: Out-of-hours
