# Supplementary material for: Out-of-hours workload among Norwegian general practitioners – an observational study
Source: BMC Health Serv Res. 2020 Oct 14;20:944. doi: 10.1186/s12913-020-05773-7 (PMC7557051; doi:10.1186/s12913-020-05773-7)
Supplement: Supplementary file 1 — Additional file 1. Qualtrics Survey [file 12913_2020_5773_MOESM1_ESM.pdf]

Nasjonalt kompetansesenter for legevaktmedisin

Intro

Fastlegers tidsbruk

Nasjonalt kompetansesenter for legevaktmedisin gjennomfører undersøkelsen på vegne av Helsedirektoratet.

Praktiske opplysninger
Det tar 2-8 minutter å fylle ut skjema for hver dag, avhengig av hvor mye forskjellig det har vært å gjøre.

Start med å fylle ut praksisprofilen din.

Du kan starte tidsregistreringen hvilken ukedag du ønsker, men det er viktig at du gjennomfører registrering for sju påfølgende dager. Vi anbefaler at du fyller ut skjemaet hver dag etter arbeidstidens slutt eller senest neste morgen hvis du har hatt kveldsarbeid eller vaktarbeid. Ved behov, bruk utskrift av huskeliste (se epostinvitasjon), døgnets regningskort og timebok til støtte for hukommelsen. Det er ikke meningen at du skal fylle ut dagsskjema mens du jobber.
Du kan gå ut av undersøkelsen ved å klikke på krysset oppe til høyre i vinduet. Gå inn igjen via lenken i e-posten. Du kommer inn der du gikk ut sist. Beveg deg frem og tilbake i undersøkelsen ved å bruke piltastene. Du kan justere feilregistreringer og foreta etterregistreringer – helt til du sender inn skjemaet den sjuende dagen.

Vi er ikke ute etter pinlig nøyaktighet, men gode anslag på hva du har brukt dagen til. Blankt felt betyr at du ikke har gjort den aktiviteten den dagen. Hvert døgn regnes fra klokken 08:00 til neste dag klokken 07:59.

Fastlege

Jobber du som fastlege i hele eller deler av tiden du deltar i undersøkelsen?

- ○ Ja, i egen fastlegehjemmel
- ○ Ja, jeg er vikar for fastlege
- ○ Nei

Takk for at du tok deg tid til å svare. Undersøkelsen er avsluttet for din del.

Fastlegers tidsbruk

1. Er du?

- ○ Mann
- ○ Kvinne

2. Alder

[▼]

3. Er du spesialist i allmennmedisin?

- ○ Ja
- ○ Nei

4. Hvor mange år har du vært fastlege?

[▼]

5. Hvor mange år har du vært allmennlege?

[▼]

6. Hvor mange innbyggere har du på din fastlegeliste?

[▼]

7. Hvor mange kurative fastlegedager har du på fastlegekontoret?

[▼]

8. Hvor mange innbyggere er det i kommunen hvor du har din fastlegepraksis?

[▼]

9. Hva er normal kjøretid fra ditt legekontor til nærmeste sykehus med akuttfunksjon?

[▼]

10. Hva er din praksisform?

11. Hvis du har kommunale oppgaver, hvor mange timer pr uke (stillingsprosent) er du ansatt for å utføre dette?
Svar kun for de oppgavene som er aktuelle for deg. Blanke felt blir stående som null tid.

Helsestasjon

Skolehelsestjeneste

Sykehjem/eldreomsorg

Kommunal ØHD

Fastlønnet legevaktlege på dagtid

Annet pasientrettet (fengsel etc.)

Adm. stilling (kommuneoverlege, legevaktsjef, smittevernlege, rådgiver)

12. Hva er din avtalte tid pr uke (stillingsprosent) for andre arbeidsforhold (eller trygdeytelser)?
Svar kun for de oppgavene som er aktuelle for deg. Blanke felt blir stående som null tid.

Forskning/undervisning

NAV, Forsvaret, Praksiskonsulent

Pensjonist, AAP, AFP, uførepensjon

Bedriftshelsetjeneste

Annet, beskriv i feltet:

Døgnregistrering i en uke

Døgnregistrering i en uke

I den neste delen ber vi deg vennligst om å registrere hvor mye tid du bruker på ulike oppgaver per døgn i løpet av en uke (mandag-søndag). Ved behov, bruk utskrift av huskeliste (se e-postinvitasjonen), døgnets regningskort og timebok til støtte for hukommelsen.

Du kan gå frem og tilbake i skjemaet mellom dagene og starte registrering for den dagen du ønsker. Vennligst registrer for alle syv dagene i uken. Når du er ferdig å registrere for en dag, kan du lukke vinduet ved å klikke på krysset oppe til høyre. Neste dag kan du åpne lenken til spørreskjemaet og fortsette der du stoppet.

Svar kun for de oppgavene som er aktuelle for deg. Blanke felt blir stående som null tid brukt.

Mandag

Mandag 08:00-Tirsdag 07:59

Vennligst registrer hvor mye tid du har brukt på ulike arbeidsoppgaver fra mandag 08:00 til tirsdag 07:59.
Svar kun for de oppgavene som er aktuelle for deg i spørsmål 1-8. Blanke felt blir stående som null tid brukt. Husk å registrere eventuelt fravær fra praksis under punkt 8. Svar på spørsmål 9 selv om du ikke har hatt pause.

Ved behov, bruk utskrift av huskeliste (se e-postinvitasjonen), døgnets regningskort og timebok til støtte for hukommelsen.

1. Fastlegepraksis

Pasientarbeid på kontoret med pasient/pårørende til stede	▼
Henvisninger, resepter, rekvisisjoner, epikriser, svar prøver og undersøkelser, journalarbeid (uten pasient til stede)	▼
Attester og erklæringer (uten pasient til stede)	▼
Dialogmøter, ansvarsgruppemøter inkl. evt. reisetid	▼
Sykebesøk inkl. reisetid	▼
Drift av praksis (IT, regnskap, møter, HMS, prosedyrer, innkjøp)	▼
Enkle kontakter, telefonkontakt og e-kommunikasjon med pasienter/pårørende	▼
Telefonkontakt og e-kommunikasjon med samarbeidspartnere (PLO-meldinger, spesialisthelsetjenesten, apotek, NAV etc.)	▼
E-konsultasjoner	▼

**2. Kommunal stilling/arbeid** (helsestasjon, skolehelsetjeneste, sykehjem, adm. stilling etc.)	▼
**3. Andre arbeidsforhold** (forskning/undervisning, NAV, Forsvaret, praksiskonsulent, BHT etc.)	▼

4. Legevakt

Tilstedevakt	▼
Hjemmevakt (inkl. aktivt pasientarbeid)	▼
Bakvakt (alle typer, inkl. turnuslegebakvakt)	▼

5. Diverse oppgaver

Tillitsvalgt, kommunale møter	▼
Veiledning, undervisning (LIS1, student, andre)	▼
Egenutdanning (lesing, nettkurs etc.)	▼
Kurs, konferanser, smågrupper, fagmøte etc.	▼

6. Totalt, hvor mye av tiden i punkt 1- 5 (praksis, henvisninger, erklæringer etc.) har du anslagsvis brukt i dag på pasienter og deres pårørende med store og sammensatte behov?
Sum tid på anslagene kan bli større enn den totale arbeidstiden, da pasienter/brukere kan høre hjemme i flere kategorier.

Kronisk syke	▼
Psykiske lidelser og rusavhengighet	▼
Skrøpelige eldre	▼
Utviklingshemning og funksjonsnedsettelser	▼

7. Totalt, hvor mye av tiden i punkt 1-5 (praksis, henvisninger, erklæringer etc.) har du anslagsvis brukt i dag på følgende grupper?

Barn og unge under 25 år ▼

Eldre over 80 år ▼

8. Midlertidig fravær denne arbeidsdagen (sykefravær, permisjon, andre årsaker) ▼

9. Hvor mye pausetid (spise- og hviletid uten arbeidsrelatert aktivitet) har du hatt i løpet av arbeidstiden dette døgnet? (beredskap, hjemmevakt og hvilende vakt regnes ikke som pause) ▼

Mandag-avslutt

Du er ferdig å registrere for mandag. Du kan nå lukke vinduet ved å klikke på krysset oppe til høyre. Svarene blir automatisk lagret. Neste dag kan du åpne lenken til spørreskjemaet og fortsette her. Klikk på pilen til høyre for å gå videre til neste dag.

Tirsdag

Tirsdag 08:00-Onsdag 07:59

Vennligst registrer hvor mye tid du har brukt på ulike arbeidsoppgaver fra tirsdag 08:00 til onsdag 07:59.
Svar kun for de oppgavene som er aktuelle for deg i spørsmål 1-8. Blanke felt blir stående som null tid brukt. Husk å registrere eventuelt fravær fra praksis under punkt 8. Svar på spørsmål 9 selv om du ikke har hatt pause.

Ved behov, bruk utskrift av huskeliste (se e-postinvitasjonen), døgnets regningskort og timebok til støtte for hukommelsen.

1. Fastlegepraksis

Pasientarbeid på kontoret med pasient/pårørende til stede ▼

Henvisninger, resepter, rekvisisjoner, epikriser, svar prøver og undersøkelser, journalarbeid (uten pasient til stede) ▼

Attester og erklæringer (uten pasient til stede) ▼

Dialogmøter, ansvarsgruppemøter inkl. evt. reisetid ▼

Sykebesøk inkl. reisetid ▼

Drift av praksis (IT, regnskap, møter, HMS, prosedyrer, innkjøp) ▼

Enkle kontakter, telefonkontakt og e-kommunikasjon med pasienter/pårørende ▼

Telefonkontakt og e-kommunikasjon med samarbeidspartnere (PLO-meldinger, spesialisthelsetjenesten, apotek, NAV etc.) ▼

E-konsultasjoner ▼

2. Kommunal stilling/arbeid (helsestasjon, skolehelsetjeneste, sykehjem, adm. stilling etc.) ▼

3. Andre arbeidsforhold (forskning/undervisning, NAV, Forsvaret, praksiskonsulent, BHT etc.) ▼

4. Legevakt

Tilstedevakt ▼

Hjemmevakt (inkl. aktivt pasientarbeid) ▼

Bakvakt (alle typer, inkl. turnuslegebakvakt) ▼

5. Diverse oppgaver

Tillitsvalgt, kommunale møter ▼

Veiledning, undervisning (LIS1, student, andre) ▼

Egenutdanning (lesing, nettkurs etc.) ▼

Kurs, konferanser, smågrupper, fagmøte etc. ▼

6. Totalt, hvor mye av tiden i punkt 1-5 (praksis, henvisninger, erklæringer etc.) har du brukt i dag på pasienter og deres pårørende med <u>store og sammensatte behov?</u>
Sum tid på anslagene kan bli større enn den totale arbeidstiden, da pasienter/brukere kan høre hjemme i flere kategorier.

Kronisk syke ▼

Psykiske lidelser og rusavhengighet ▼

Skrøpelige eldre ▼

Utviklingshemning og funksjonsnedsettelser ▼

7. Totalt, hvor mye av tiden i punkt 1-5 (praksis, henvisninger, erklæringer etc.) har du anslagsvis brukt i dag på følgende grupper?

Barn og unge under 25 år ▼

Eldre over 80 år ▼

8. Midlertidig fravær denne arbeidsdagen (sykefravær, permisjon, andre årsaker) ▼

9. Hvor mye pausetid (spise- og hviletid uten arbeidsrelatert aktivitet) har du hatt i løpet av arbeidstiden dette døgnet? (beredskap, hjemmevakt og hvilende vakt regnes ikke som pause)

Tirsdag-avslutt

Du er ferdig å registrere for tirsdag. Du kan nå lukke vinduet ved å klikke på krysset oppe til høyre. Svarene blir automatisk lagret. Neste dag kan du åpne lenken til spørreskjemaet og fortsette her. Klikk på pilen til høyre for å gå videre til neste dag.

Onsdag

Onsdag 08:00-Torsdag 07:59

Vennligst registrer hvor mye tid du har brukt på ulike arbeidsoppgaver fra onsdag 08:00 til torsdag 07:59.
Svar kun for de oppgavene som er aktuelle for deg i spørsmål 1-8. Blanke felt blir stående som null tid brukt. Husk å registrere eventuelt fravær fra praksis under punkt 8. Svar på spørsmål 9 selv om du ikke har hatt pause.

Ved behov, bruk utskrift av huskeliste (se e-postinvitasjonen), døgnets regningskort og timebok til støtte for hukommelsen.

1. Fastlegepraksis

Pasientarbeid på kontoret med pasient/pårørende til stede

Henvisninger, resepter, rekvisisjoner, epikriser, svar prøver og undersøkelser, journalarbeid (uten pasient til stede)

Attester og erklæringer (uten pasient til stede)

Dialogmøter, ansvarsgruppemøter inkl. evt. reisetid

Sykebesøk inkl. reisetid

Drift av praksis (IT, regnskap, møter, HMS, prosedyrer, innkjøp)

Enkle kontakter, telefonkontakt og e-kommunikasjon med pasienter/pårørende

Telefonkontakt og e-kommunikasjon med samarbeidspartnere (PLO-meldinger, spesialisthelsetjenesten, apotek, NAV etc.)

E-konsultasjoner

2. Kommunal stilling/arbeid (helsestasjon, skolehelsetjeneste, sykehjem, adm. stilling etc.)

3. Andre arbeidsforhold (forskning/undervisning, NAV, Forsvaret, praksiskonsulent, BHT etc.)

4. Legevakt

Tilstedevakt

Hjemmevakt (inkl. aktivt pasientarbeid)

Bakvakt (alle typer, inkl. turnuslegebakvakt)

5. Diverse oppgaver

Tillitsvalgt, kommunale møter ▼

Veiledning, undervisning (LIS1, student, andre) ▼

Egenutdanning (lesing, nettkurs etc.) ▼

Kurs, konferanser, smågrupper, fagmøte etc. ▼

6. Totalt, hvor mye av tiden i punkt 1-5 (praksis, henvisninger, erklæringer etc.) har du brukt i dag på pasienter og deres pårørende med <u>store og sammensatte behov?</u>
Sum tid på anslagene kan bli større enn den totale arbeidstiden, da pasienter/brukere kan høre hjemme i flere kategorier.

Kronisk syke ▼

Psykiske lidelser og rusavhengighet ▼

Skrøpelige eldre ▼

Utviklingshemning og funksjonsnedsettelser ▼

7. Totalt, hvor mye av tiden i punkt 1-5 (praksis, henvisninger, erklæringer etc.) har du anslagsvis brukt i dag på følgende grupper?

Barn og unge under 25 år ▼

Eldre over 80 år ▼

8. Midlertidig fravær denne arbeidsdagen (sykefravær, permisjon, andre årsaker) ▼

9. Hvor mye pausetid (spise- og hviletid uten arbeidsrelatert aktivitet) har du hatt i løpet av arbeidstiden dette døgnet? (beredskap, hjemmevakt og hvilende vakt regnes ikke som pause) ▼

Onsdag-avslutt

Du er ferdig å registrere for onsdag. Du kan nå lukke vinduet ved å klikke på krysset oppe til høyre. Svarene blir automatisk lagret. Neste dag kan du åpne lenken til spørreskjemaet og fortsette her. Klikk på pilen til høyre for å gå videre til neste dag.

Torsdag

Torsdag 08:00-Fredag 07:59

Vennligst registrer hvor mye tid du har brukt på ulike arbeidsoppgaver fra torsdag 08:00 til fredag 07:59.
Svar kun for de oppgavene som er aktuelle for deg i spørsmål 1-8. Blanke felt blir stående som null tid brukt. Husk å registrere eventuelt fravær fra praksis under punkt 8. Svar på spørsmål 9 selv om du ikke har hatt pause.

Ved behov, bruk utskrift av huskeliste (se e-postinvitasjonen), døgnets regningskort og timebok til støtte for hukommelsen.

1. Fastlegepraksis

Pasientarbeid på kontoret med pasient/pårørende til stede	▼
Henvisninger, resepter, rekvisisjoner, epikriser, svar prøver og undersøkelser, journalarbeid (uten pasient til stede)	▼
Attester og erklæringer (uten pasient til stede)	▼
Dialogmøter, ansvarsgruppemøter inkl. evt. reisetid	▼
Sykebesøk inkl. reisetid	▼
Drift av praksis (IT, regnskap, møter, HMS, prosedyrer, innkjøp)	▼
Enkle kontakter, telefonkontakt og e-kommunikasjon med pasienter/pårørende	▼
Telefonkontakt og e-kommunikasjon med samarbeidspartnere (PLO-meldinger, spesialisthelsetjenesten, apotek, NAV etc.)	▼
E-konsultasjoner	▼

**2. Kommunal stilling/arbeid** (helsestasjon, skolehelsetjeneste, sykehjem, adm. stilling etc.)	▼
**3. Andre arbeidsforhold** (forskning/undervisning, NAV, Forsvaret, praksiskonsulent, BHT etc.)	▼

4. Legevakt

Tilstedevakt	▼
Hjemmevakt (inkl. aktivt pasientarbeid)	▼
Bakvakt (alle typer, inkl. turnuslegebakvakt)	▼

5. Diverse oppgaver

Tillitsvalgt, kommunale møter	▼
Veiledning, undervisning (LIS1, student, andre)	▼
Egenutdanning (lesing, nettkurs etc.)	▼
Kurs, konferanser, smågrupper, fagmøte etc.	▼

6. Totalt, hvor mye av tiden i punkt 1-5 (praksis, henvisninger, erklæringer etc.) har du brukt i dag på pasienter og deres pårørende med <u>store og sammensatte behov?</u>

Sum tid på anslagene kan bli større enn den totale arbeidstiden, da pasienter/brukere kan høre hjemme i flere kategorier.

Kronisk syke
Psykiske lidelser og rusavhengighet
Skrøpelige eldre
Utviklingshemning og funksjonsnedsettelser

7. Totalt, hvor mye av tiden i punkt 1-5 (praksis, henvisninger, erklæringer etc.) har du anslagsvis brukt i dag på følgende grupper?

Barn og unge under 25 år
Eldre over 80 år

8. Midlertidig fravær denne arbeidsdagen (sykefravær, permisjon, andre årsaker)

9. Hvor mye pausetid (spise- og hviletid uten arbeidsrelatert aktivitet) har du hatt i løpet av arbeidstiden dette døgnet? (beredskap, hjemmevakt og hvilende vakt regnes ikke som pause)

Torsdag-avslutt

Du er ferdig å registrere for torsdag. Du kan nå lukke vinduet ved å klikke på krysset oppe til høyre. Svarene blir automatisk lagret. Neste dag kan du åpne lenken til spørreskjemaet og fortsette her. Klikk på pilen til høyre for å gå videre til neste dag.

Fredag

Fredag 08:00-Lørdag 07:59

Vennligst registrer hvor mye tid du har brukt på ulike arbeidsoppgaver fra fredag 08:00 til lørdag 07:59.
Svar kun for de oppgavene som er aktuelle for deg i spørsmål 1-8. Blanke felt blir stående som null tid brukt. Husk å registrere eventuelt fravær fra praksis under punkt 8. Svar på spørsmål 9 selv om du ikke har hatt pause.

Ved behov, bruk utskrift av huskeliste (se e-postinvitasjonen), døgnets regningskort og timebok til støtte for hukommelsen.

1. Fastlegepraksis

Pasientarbeid på kontoret med pasient/pårørende til stede
Henvisninger, resepter, rekvisisjoner, epikriser, svar prøver og undersøkelser, journalarbeid (uten pasient til stede)
Attester og erklæringer (uten pasient til stede)
Dialogmøter, ansvarsgruppemøter inkl. evt. reisetid
Sykebesøk inkl. reisetid

Drift av praksis (IT, regnskap, møter, HMS, prosedyrer, innkjøp)

Enkle kontakter, telefonkontakt og e-kommunikasjon med pasienter/pårørende

Telefonkontakt og e-kommunikasjon med samarbeidspartnere (PLO-meldinger, spesialisthelsetjenesten, apotek, NAV etc.)

E-konsultasjoner

2. Kommunal stilling/arbeid (helsestasjon, skolehelsetjeneste, sykehjem, adm. stilling etc.)

3. Andre arbeidsforhold (forskning/undervisning, NAV, Forsvaret, praksiskonsulent, BHT etc.)

4. Legevakt

Tilstedevakt

Hjemmevakt (inkl. aktivt pasientarbeid)

Bakvakt (alle typer, inkl. turnuslegebakvakt)

5. Diverse oppgaver

Tillitsvalgt, kommunale møter

Veiledning, undervisning (LIS1, student, andre)

Egenutdanning (lesing, nettkurs etc.)

Kurs, konferanser, smågrupper, fagmøte etc.

6. Totalt, hvor mye av tiden i punkt 1-5 (praksis, henvisninger, erklæringer etc.) har du brukt i dag på pasienter og deres pårørende med store og sammensatte behov?

Sum tid på anslagene kan bli større enn den totale arbeidstiden, da pasienter/brukere kan høre hjemme i flere kategorier.

Kronisk syke

Psykiske lidelser og rusavhengighet

Skrøpelige eldre

Utviklingshemning og funksjonsnedsettelser

7. Totalt, hvor mye av tiden i punkt 1-5 (praksis, henvisninger, erklæringer etc.) har du anslagsvis brukt i dag på følgende grupper?

Barn og unge under 25 år

Eldre over 80 år

8. Midlertidig fravær denne arbeidsdagen (sykefravær, permisjon, andre årsaker) ▼

9. Hvor mye pausetid (spise- og hviletid uten arbeidsrelatert aktivitet) har du hatt i løpet av arbeidstiden dette døgnet? (beredskap, hjemmevakt og hvilende vakt regnes ikke som pause) ▼

Fredag-avslutt

Du er ferdig å registrere for fredag. Du kan nå lukke vinduet ved å klikke på krysset oppe til høyre. Svarene blir automatisk lagret. Neste dag kan du åpne lenken til spørreskjemaet og fortsette her. Klikk på pilen til høyre for å gå videre til neste dag.

Lørdag

Lørdag 08:00-Søndag 07:59

Vennligst registrer hvor mye tid du har brukt på ulike arbeidsoppgaver fra lørdag 08:00 til søndag 07:59.
Svar kun for de oppgavene som er aktuelle for deg. Blanke felt blir stående som null tid brukt.

Ved behov, bruk utskrift av huskeliste (se e-postinvitasjonen), døgnets regningskort og timebok til støtte for hukommelsen.

1. Fastlegepraksis

Pasientarbeid på kontoret med pasient/påprørende til stede	▼
Henvisninger, resepter, rekvisisjoner, epikriser, svar prøver og undersøkelser, journalarbeid (uten pasient til stede)	▼
Attester og erklæringer (uten pasient til stede)	▼
Dialogmøter, ansvarsgruppemøter inkl. evt. reisetid	▼
Sykebesøk inkl. reisetid	▼
Drift av praksis (IT, regnskap, møter, HMS, prosedyrer, innkjøp)	▼
Enkle kontakter, telefonkontakt og e-kommunikasjon med pasienter/pårørende	▼
Telefonkontakt og e-kommunikasjon med samarbeidspartnere (PLO-meldinger, spesialisthelsetjenesten, apotek, NAV etc.)	▼
E-konsultasjoner	▼

2. Kommunal stilling/arbeid (helsestasjon, skolehelsetjeneste, sykehjem, adm. stilling etc.) ▼

3. Andre arbeidsforhold (forskning/undervisning, NAV, Forsvaret, praksiskonsulent, BHT etc.) ▼

4. Legevakt

Tilstedevakt

Hjemmevakt (inkl. aktivt pasientarbeid)

Bakvakt (alle typer, inkl. turnuslegebakvakt)

5. Diverse oppgaver

Tillitsvalgt, kommunale møter

Veiledning, undervisning (LIS1, student, andre)

Egenutdanning (lesing, nettkurs etc.)

Kurs, konferanser, smågrupper, fagmøte etc.

6. Totalt, hvor mye av tiden i punkt 1-5 (praksis, henvisninger, erklæringer etc.) har du brukt i dag på pasienter og deres pårørende med store og sammensatte behov?

Sum tid på anslagene kan bli større enn den totale arbeidstiden, da pasienter/brukere kan høre hjemme i flere kategorier.

Kronisk syke

Psykiske lidelser og rusavhengighet

Skrøpelige eldre

Utviklingshemning og funksjonsnedsettelser

7. Totalt, hvor mye av tiden i punkt 1-5 (praksis, henvisninger, erklæringer etc.) har du anslagsvis brukt i dag på følgende grupper?

Barn og unge under 25 år

Eldre over 80 år

8. Midlertidig fravær denne arbeidsdagen (sykefravær, permisjon, andre årsaker)

9. Hvor mye pausetid (spise- og hviletid uten arbeidsrelatert aktivitet) har du hatt i løpet av arbeidstiden dette døgnet? (beredskap, hjemmevakt og hvilende vakt regnes ikke som pause)

Lørdag-avslutt

Du er ferdig å registrere for lørdag. Du kan nå lukke vinduet ved å klikke på krysset oppe til høyre. Svarene blir automatisk lagret. Neste dag kan du åpne lenken til spørreskjemaet og fortsette her. Klikk på pilen til høyre for å gå videre til neste dag.

Søndag

Søndag 08:00-Mandag 07:59

Vennligst registrer hvor mye tid du har brukt på ulike arbeidsoppgaver fra søndag 08:00 til mandag 07:59.
Svar kun for de oppgavene som er aktuelle for deg. Blanke felt blir stående som null tid brukt.

Ved behov, bruk utskrift av huskeliste (se e-postinvitasjonen), døgnets regningskort og timebok til støtte for hukommelsen.

1. Fastlegepraksis

Pasientarbeid på kontoret med pasient/pårørende til stede ▼

Henvisninger, resepter, rekvisisjoner, epikriser, svar prøver og undersøkelser, journalarbeid (uten pasient til stede) ▼

Attester og erklæringer (uten pasient til stede) ▼

Dialogmøter, ansvarsgruppemøter inkl. evt. reisetid ▼

Sykebesøk inkl. reisetid ▼

Drift av praksis (IT, regnskap, møter, HMS, prosedyrer, innkjøp) ▼

Enkle kontakter, telefonkontakt og e-kommunikasjon med pasienter/pårørende ▼

Telefonkontakt og e-kommunikasjon med samarbeidspartnere (PLO-meldinger, spesialisthelsetjenesten, apotek, NAV etc.) ▼

E-konsultasjoner ▼

2. Kommunal stilling/arbeid (helsestasjon, skolehelsetjeneste, sykehjem, adm. stilling etc.) ▼

3. Andre arbeidsforhold (forskning/undervisning, NAV, Forsvaret, praksiskonsulent, BHT etc.) ▼

4. Legevakt

Tilstedevakt ▼

Hjemmevakt (inkl. aktivt pasientarbeid) ▼

Bakvakt (alle typer, inkl. turnuslegebakvakt) ▼

5. Diverse oppgaver

Tillitsvalgt, kommunale møter ▼

Veiledning, undervisning (LIS1, student, andre) ▼

Egenutdanning (lesing, nettkurs etc.) ▼

Kurs, konferanser, smågrupper, fagmøte etc.

6. Totalt, hvor mye av tiden i punkt 1-5 (praksis, henvisninger, erklæringer etc.) har du brukt i dag på pasienter og deres pårørende med store og sammensatte behov?
Sum tid på anslagene kan bli større enn den totale arbeidstiden, da pasienter/brukere kan høre hjemme i flere kategorier.

Kronisk syke

Psykiske lidelser og rusavhengighet

Skrøpelige eldre

Utviklingshemning og funksjonsnedsettelser

7. Totalt, hvor mye av tiden i punkt 1-5 (praksis, henvisninger, erklæringer etc.) har du anslagsvis brukt i dag på følgende grupper?

Barn og unge under 25 år

Eldre over 80 år

8. Midlertidig fravær denne arbeidsdagen (sykefravær, permisjon, andre årsaker)

9. Hvor mye pausetid (spise- og hviletid uten arbeidsrelatert aktivitet) har du hatt i løpet av arbeidstiden dette døgnet? (beredskap, hjemmevakt og hvilende vakt regnes ikke som pause)

Søndag-avslutt

Du er ferdig å registrere for søndag. Svarene blir automatisk lagret. Gå videre for å avslutte.

Send

Dersom du er ferdig å registrere og vil sende inn ditt svar, klikk send.
Dersom du ikke er ferdig, gå tilbake i undersøkelsen og fullfør.
